# Left Paraduodenal Hernia: A Challenging Diagnosis in Intestinal Obstruction

**DOI:** 10.7759/cureus.83540

**Published:** 2025-05-05

**Authors:** Bashar I Alshdaifat, Deema M Alhayali, Omar S Alani, Hazim I Al Sane, Yaser A Rasmi

**Affiliations:** 1 General Surgery, Al Qassimi Hospital, Sharjah, ARE; 2 College of Medicine, University of Sharjah, Sharjah, ARE

**Keywords:** compartment syndrome, hyperactive bowel, internal hernia, paraduodenal hernia, small bowel obstruction

## Abstract

Internal hernias are a rare cause of intestinal obstruction, accounting for a small proportion of cases. Among these, paraduodenal hernias represent a significant subtype and require prompt recognition and intervention to prevent life-threatening complications. We report the case of a 31-year-old male with no significant medical or surgical history who presented with a three-day history of generalized abdominal pain, vomiting, and constipation. Clinical examination revealed abdominal distension, tenderness, and hyperactive bowel sounds. Laboratory findings were unremarkable except for leukocytosis. Abdominal X-ray demonstrated features of small bowel obstruction, and CT imaging identified findings suggestive of a left paraduodenal hernia. Exploratory laparotomy revealed a large encapsulated peritoneal sac originating from the left paraduodenal region and extending into the pelvis. Dilated and edematous but viable small bowel loops were released, and adhesiolysis was performed. A planned second-look surgery two days later confirmed resolution of bowel edema, and the abdomen was closed without complications. Paraduodenal hernias arise from a congenital anomaly involving the mesentery and often present as intermittent or acute bowel obstruction. Diagnosis is challenging due to nonspecific symptoms but can be facilitated by CT imaging. Definitive management involves surgical reduction and repair of the hernia, whether through open or laparoscopic approaches. This case highlights the importance of considering internal hernias in young patients with small bowel obstruction and no prior abdominal surgery. Prompt imaging, timely surgery, and individualized management are crucial for optimal outcomes.

## Introduction

Internal hernia is a rare but potentially life-threatening cause of intestinal obstruction, accounting for 5% to 8% of small bowel obstructions [1,2]. Paraduodenal hernia, which involves herniation of the small bowel through a congenital opening in the mesentery, accounts for the majority of internal hernia cases (30% to 53%). Notably, left paraduodenal hernia is three times more common than the right [3]. Other, less common types of internal hernias include pericecal, transmesenteric, transmesocolic, transomental, and others [4].

Regardless of the type, internal hernias present a diagnostic challenge due to their variable clinical presentations, which may range from recurrent abdominal pain to acute bowel obstruction. In this report, we present the case of a 31-year-old patient with intestinal obstruction who presented with acute abdominal pain and vomiting. A CT scan revealed signs of small bowel obstruction, with a left paraduodenal hernia identified as the likely cause. The patient underwent exploratory laparotomy, which revealed a large encapsulated peritoneal sac extending from the paraduodenal region to the pelvis, containing distended small bowel loops.

We emphasize the critical importance of considering internal hernia as a potential cause of intestinal obstruction, given its high risk of strangulation and ischemia if left untreated.

## Case presentation

A previously healthy 31-year-old male patient presented to the ED with a 3-day history of worsening abdominal pain, more severe in the lower abdomen. The pain began suddenly after he woke up and was associated with multiple episodes of yellow vomit, triggered by oral intake. The patient initially sought medical care on the first day of his symptoms and was managed symptomatically at another hospital, but he did not respond well to treatment. He presented to our hospital on day 3 of his symptoms with increased pain and vomiting, in addition to constipation, his last bowel movement had occurred three days prior to presentation, though he was still able to pass flatus. His past medical history was notable only for peptic ulcer disease, diagnosed by endoscopy and successfully treated three years ago. He denied any similar previous episodes of abdominal pain. His past surgical history was unremarkable, with no prior surgeries.

Upon physical examination, the patient was awake, alert, and oriented. He appeared to be in pain but was hemodynamically stable, with a heart rate of 78 bpm, blood pressure of 133/78 mmHg, SpO₂ of 100%, and he was afebrile. There were no signs of peritonitis and no palpable mass, which made the diagnosis more challenging. The patient had a normal body build but appeared dehydrated. Cardiovascular and respiratory examinations were unremarkable, with normal heart sounds and vesicular breath sounds bilaterally. On abdominal examination, the abdomen was severely distended and tense, with tenderness mostly localized to the lower abdomen. No surgical scars were observed, and no organomegaly was noted on palpation. Auscultation revealed hyperactive bowel sounds, and per rectal examination was unremarkable.

Blood tests revealed the following: hemoglobin of 13.9 g/dL (normal), white blood cell count of 15.83/mL (elevated), and platelet count of 277,000/mL (normal). C-reactive protein (CRP) was 3.4 mg/L (slightly elevated). Electrolytes, urea, liver function tests, platelets, and coagulation profile were all within normal limits. The elevated white blood cell count was consistent with a stress response or early inflammation, while the slightly elevated CRP suggested the absence of perforation, necrosis, or systemic inflammatory or ischemic complications, as CRP would typically be markedly elevated in such conditions.

**Figure 1:**
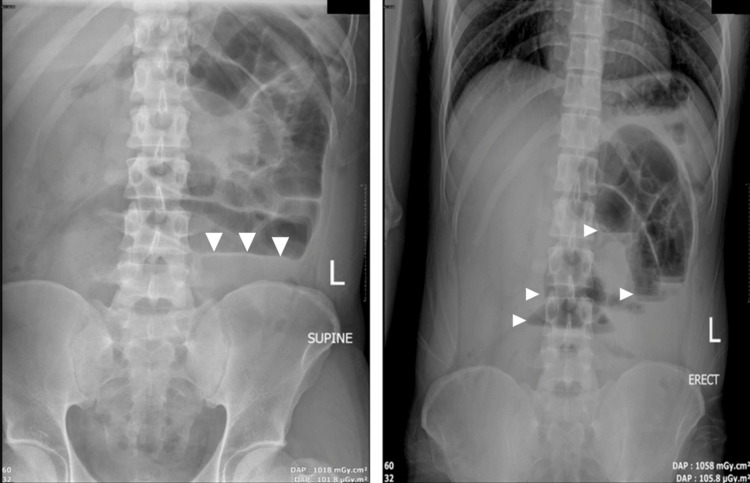
Abdominal X-ray (supine and erect).

An abdominal X-ray was performed and showed multiple air-fluid levels in the proximal small bowel. No free intra-abdominal air was seen (Figure 1).

**Figure 2:**
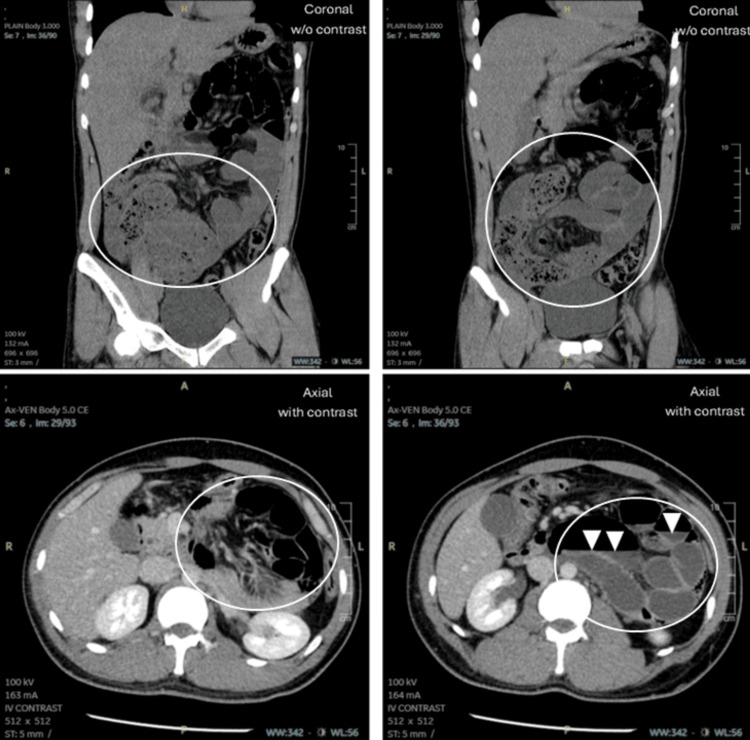
CT scan of the abdomen (coronal and axial views).

A CT scan with and without contrast was performed on the abdomen and pelvis. The scan revealed changes consistent with small bowel obstruction, primarily involving the ileum and distal jejunal loops. The transitional point appeared to be at the terminal ileum, which was collapsed, leading to dilation of the proximal small bowel, measuring up to 4 cm in diameter. Additionally, multiple air-fluid levels and a small bowel feces sign were observed in the distal ileum. The large bowel was normal in diameter. A portion of the jejunum was found within the lesser sac, although these loops were not significantly dilated. Findings were suggestive of a left paraduodenal hernia, and orally ingested contrast was not seen passing into those distal bowel loops. There was no evidence of mural thickening or abnormal enhancement, which suggests that the bowel was viable. However, the presence of these signs contributed to diagnostic uncertainty, as they can complicate the identification of a left paraduodenal hernia. No pneumoperitoneum was seen. Additionally, there were no signs of appendicitis (Figure 2).

**Figure 3:**
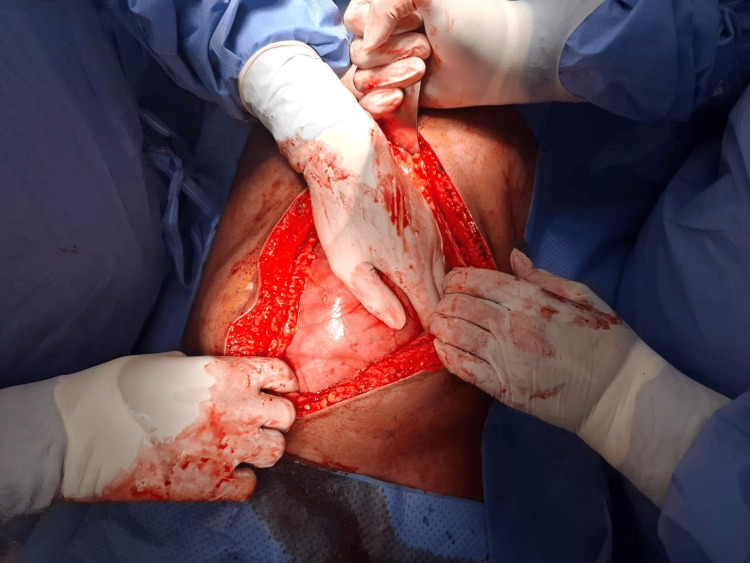
Intraoperative findings: revealing the large encapsulated hernia sac.

**Figure 4:**
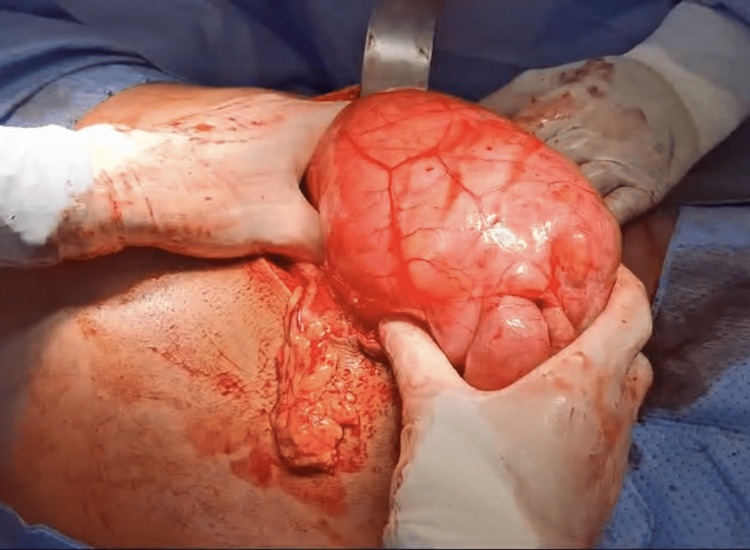
Intraoperative findings: the large encapsulated hernia sac.

**Figure 5:**
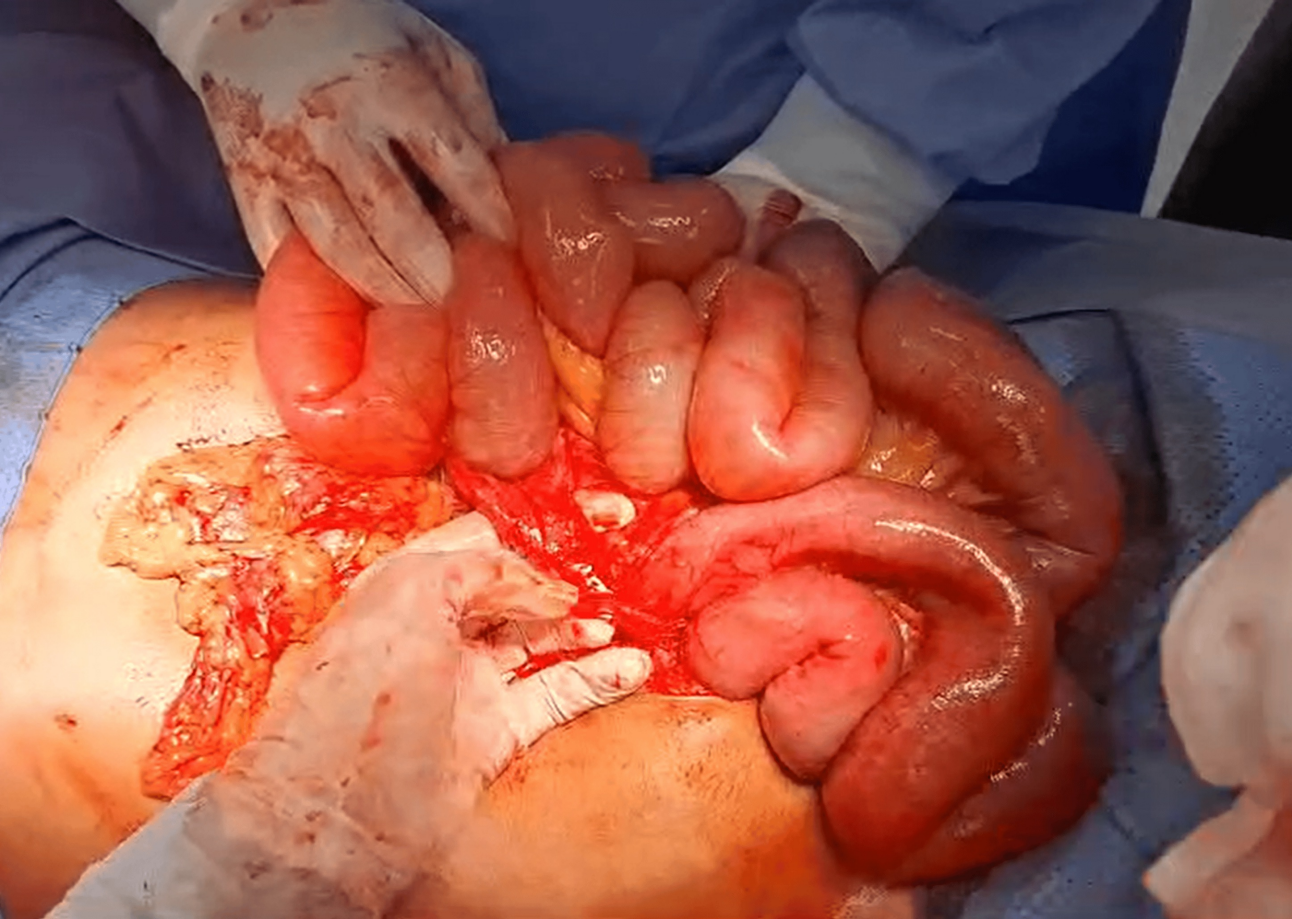
Intraoperative findings: dilated small bowel loop.

**Figure 6:**
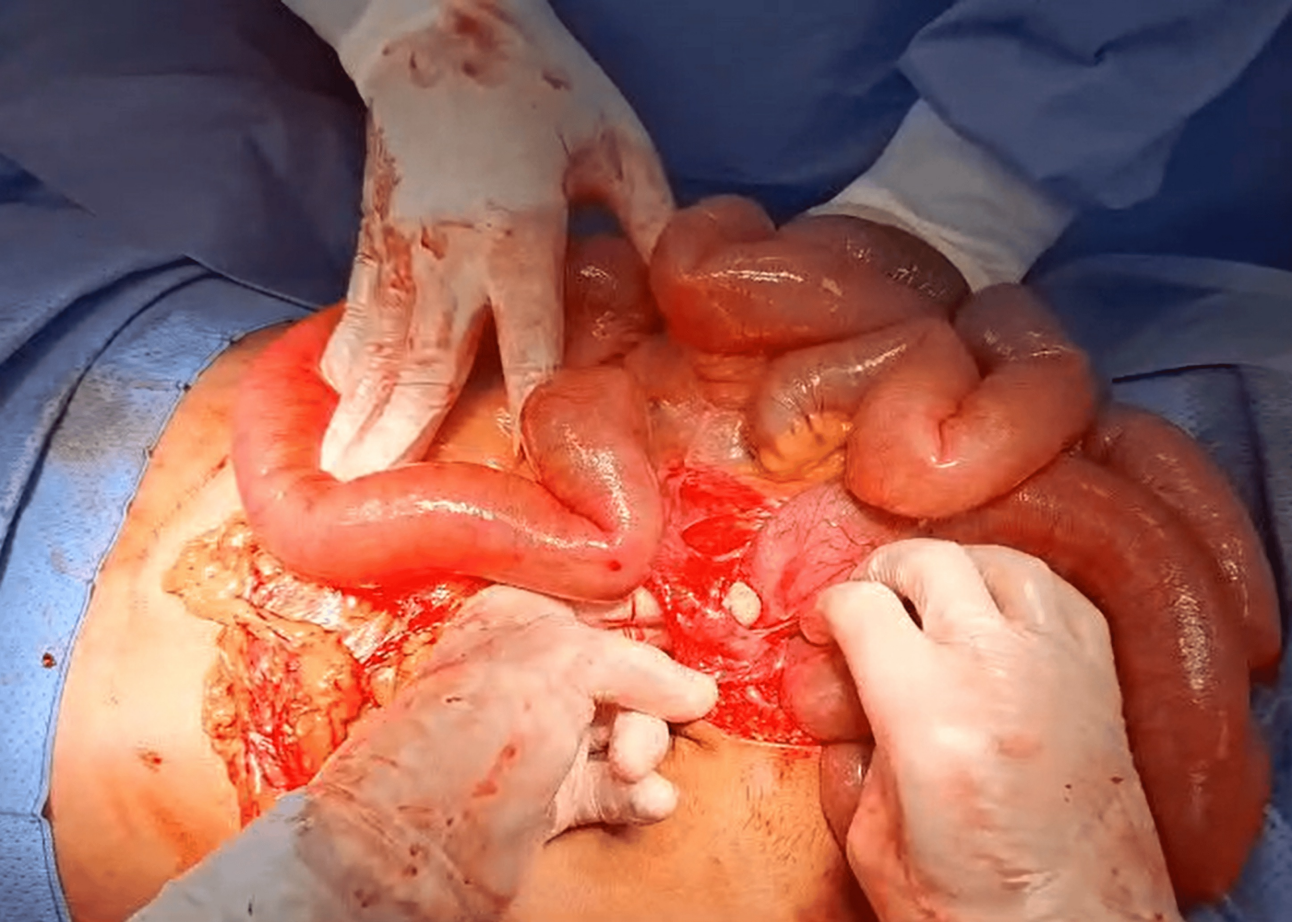
Intraoperative findings: adhesions with the ascending colon.

Based on the clinical presentation and radiological findings, the patient underwent an exploratory laparotomy, as the abdomen was severely distended and a laparoscopic approach could not be performed. In the operating room, the patient was placed in a supine position under general anesthesia. A midline laparotomy incision was made, and the abdominal layers were opened, revealing a large encapsulated peritoneal sac extending from the left paraduodenal area to the pelvis (Figures 3-4). The sac was excised and opened, revealing dilated, edematous small bowel loops, which appeared viable upon inspection (Figure 5). The large bowel was collapsed, and adhesions between the sac and the large bowel were noted (Figure 6). The small bowel was released from the peritoneal sac, and adhesiolysis was performed. The hernia defect was not repaired during the surgery, as this was deemed appropriate by the surgical team based on clinical judgment, taking into account factors such as the patient’s condition and anatomical considerations. The abdomen was irrigated with normal saline, and a drain was placed in the pelvis. Due to the risk of compartment syndrome, given the distended and edematous small bowel, the abdomen was initially left open with a temporary closure using an Ioban dressing, with a planned second-look procedure.

Two days later, the patient underwent a second exploratory laparotomy. On re-exploration, the bowel appeared healthy, with resolution of edema and distension. The abdomen was washed with normal saline, and closure was performed in layers; the fascial layer was closed using a loop suture, and the skin was closed with staples. The patient tolerated the surgery well and had no complaints. Postoperatively, he was monitored in the surgical ward and recovered without complications.

## Discussion

An internal hernia occurs when an organ or a segment of the intestine protrudes through a congenital or abnormal opening within the peritoneum or mesentery, forming an enclosed space within the peritoneal cavity. Paraduodenal hernias represent distinct types based on anatomical location and their unique embryologic development [5]. Since they are often asymptomatic, paraduodenal hernias are typically diagnosed incidentally or during emergency presentations with bowel obstruction [6].

Left paraduodenal hernias are congenital anomalies that occur due to abnormal intestinal rotation, wherein the small intestine herniates into an avascular region of the transverse-descending mesocolon. This results in the formation of a hernial sac within Landzert’s fossa, located to the left of the fourth portion of the duodenum, posterior to the inferior mesenteric vein and the left branches of the middle colic artery [7]. Loops of the small bowel, typically the jejunum, prolapse posteroinferiorly through Landzert’s fossa into the left segment of the transverse mesocolon, resulting in entrapment within the mesenteric sac [1].

Paraduodenal hernias have a varied and nonspecific presentation, ranging from lifelong asymptomatic cases to recurrent upper abdominal pain (43%) or acute small bowel obstruction. In severe cases, a left paraduodenal hernia may present as a palpable abdominal mass in the left upper quadrant, accompanied by dilation of eccentrically positioned ileal loops at the hernia site [8]. The symptoms presented by this patient, including intermittent abdominal pain, vomiting, and constipation, align with common presentations of paraduodenal hernias in the literature. This patient’s progression from intermittent symptoms to complete obstruction is consistent with the natural history of left paraduodenal hernias, which can evolve from asymptomatic to acutely symptomatic depending on the degree of bowel entrapment. Many patients report nonspecific symptoms until acute episodes occur, often due to bowel obstruction.

Paraduodenal hernias are frequently diagnosed incidentally during laparotomy, autopsy, or radiological imaging conducted for unrelated conditions [9]. Preoperative diagnosis of an asymptomatic paraduodenal hernia is challenging, as imaging is often unremarkable unless performed during a symptomatic episode [10]. On X-ray, paraduodenal hernias are traditionally seen as clustered small bowel loops in the upper right or left quadrants. However, CT has become the preferred method for diagnosing internal hernias, although plain radiographs and barium follow-through studies can also aid in diagnosis [5].

Abdominal CT is the gold standard for diagnosing left paraduodenal hernia, revealing several characteristic radiological signs associated with the condition. These may include a “cluster” of small bowel loops, an encapsulated mass near the ligament of Treitz, depression of the duodenojejunal junction, and a mass effect on the posterior stomach wall. Additional features often observed are congestion and crowding of the mesenteric vessels, rightward displacement of the main mesenteric trunk, anterocephalad displacement of the inferior mesenteric vein (defining the hernial defect), and depression of the transverse colon [6,8].

The treatment approach for paraduodenal hernias follows the standard principles of hernia surgery, which include reduction of the herniated contents, excision of the hernia sac, restoration of normal bowel anatomy, and repair of the defect. If the hernia opening is sufficiently large, manual reduction of the small intestine may be possible, and the defect can be closed using nonabsorbable sutures. In cases where the small bowel is edematous, the hernia orifice is constricted, or adhesions within the sac prevent reduction, the orifice can be widened by excising the avascular plane to the right of the inferior mesenteric vessels [4,11]. Once diagnosed, paraduodenal hernias should be surgically repaired, as up to 50% of cases result in intestinal obstruction [12]. While recurrence is rare, ongoing follow-up with clinical monitoring, and, in some cases, imaging, may be recommended to assess for complications.

## Conclusions

In conclusion, this case highlights the importance of considering internal hernias, particularly paraduodenal hernias, as potential causes of small bowel obstruction in young, otherwise healthy adults with no history of abdominal surgery. Prompt diagnosis through imaging and timely surgical intervention are crucial to prevent complications such as bowel ischemia or perforation. Additionally, this case underscores the benefits of a staged surgical approach in patients with significant bowel distension, as it helps reduce the risk of compartment syndrome and supports optimal recovery.
